# Bactericidal Effects of Diode Laser Irradiation on *Enterococcus faecalis* Using Periapical Lesion Defect Model

**DOI:** 10.5402/2011/870364

**Published:** 2011-07-14

**Authors:** Masato Nagayoshi, Tatsuji Nishihara, Keisuke Nakashima, Shigetsugu Iwaki, Ker-Kong Chen, Masamichi Terashita, Chiaki Kitamura

**Affiliations:** ^1^Division of Pulp Biology, Operative Dentistry, and Endodontics, Department of Cariology and Periodontology, Kyushu Dental College, 2-6-1 Manazuru, Kokurakita, Kitakyushu 803-8580, Japan; ^2^Division of Infections and Molecular Biology, Department of Health Promotion, Kyushu Dental College, 2-6-1 Manazuru, Kokurakita, Kitakyushu 803-8580, Japan; ^3^Division of Periodontology, Department of Cariology and Periodontology, Kyushu Dental College, 2-6-1 Manazuru, Kokurakita, Kitakyushu 803-8580, Japan; ^4^Marketing Research Section, R&D Department, NISSIN DENTAL PRODUCTS INC., 22-1 miyabayashi, asahi, Kameoka, Kyoto 621-0001, Japan; ^5^Department of Conservative Dentistry, Kaohsiung Medical University Hospital and College of Dental Medicine, Kaohsiung Medical University, Shih-Chuan 1st Road, Kaohsiung 80708, Taiwan; ^6^Division of Comprehensive Dentistry, Department of Clinical Communication and Practice, Kyushu Dental College, 2-6-1 Manazuru, Kokurakita, Kitakyushu 803-8580, Japan

## Abstract

*Objective*. Photodynamic therapy has been expanded for use in endodontic treatment. The aim of this study was to investigate the antimicrobial effects of diode laser irradiation on endodontic pathogens in periapical lesions using an *in vitro* apical lesion model. *Study Design*. *Enterococcus faecalis* in 0.5% semisolid agar with a photosensitizer was injected into apical lesion area of *in vitro* apical lesion model. The direct effects of irradiation with a diode laser as well as heat produced by irradiation on the viability of microorganisms in the lesions were analyzed. *Results*. The viability of *E. faecalis* was significantly reduced by the combination of a photosensitizer and laser irradiation. The temperature caused by irradiation rose, however, there were no cytotoxic effects of heat on the viability of *E. faecalis*. *Conclusion*. Our results suggest that utilization of a diode laser in combination with a photosensitizer may be useful for clinical treatment of periapical lesions.

## 1. Introduction

It is generally accepted that disinfecting processes are essential for successful root canal treatment, and antimicrobial irrigants to remove microorganisms are important for chemomechanical preparation of a root canal [1, 2]. Endodontic irrigants are required to have a broad spectrum of antimicrobial activities, as well as a relative lack of toxicity against sound periapical tissue. Sodium hypochlorite (NaOCl) is a major endodontic irrigant, however, it has cytotoxic and neurotoxic effects when extruded into periapical tissues [3, 4]. To develop a safe endodontic irrigants, ozonated water was previously examined as an endodontic irrigant and demonstrated to be a useful irrigant for removal of microorganisms from root canals without damage to other tissues [5]. However, it is also known that complete elimination of microorganisms from root canals and periapical lesions by antimicrobial irrigants only is difficult, because of the anatomical complexities of root canals, deep invasion of microorganisms into dentinal tubules, and formation of biofilms on the surface of root apex, resulting in persistent apical periodontitis [6–8]. In addition, several studies have reported that the lack of response of refractory periapical lesion is due to the in inaccessibility of the extraradicular microorganisms or to the presence of microorganisms [9–11]. 

 Recently, photodynamic antimicrobial chemotherapy has received focus as an alternate antibacterial, antifungal, and antiviral treatment for drug-resistant microorganisms [12, 13]. Along that line, disinfection of root canals by laser irradiation has been demonstrated [14–16]. When using laser irradiation for disinfection of root canals and periapical lesions, damage to periapical tissues by heat produced by the irradiation procedure should be avoided [17–19]. Several studies have reported the antimicrobial effectiveness and safety of laser irradiation of root canals. However, there are no known reports regarding the antimicrobial effects of laser irradiation on microorganisms in periapical lesions without corresponding heat damage to sound periapical tissues, because of difficulties in mimicking the related environment.

 A few studies have reported that use of a photosensitizer in combination with laser irradiation was effective for selective elimination of microorganisms from root canals and periapical lesions [20–22]. The aim of this study was to investigate the antimicrobial effects of diode laser irradiation in combination with a photosensitizer against *Enterococcus faecalis*, one of the major organisms related to persistent apical periodontitis [10, 11, 23, 24] in periapical lesions using an *in vitro* apical lesion model. In addition, irradiation-induced heat changes and the effects of heat on *E. faecalis* were examined using our periapical lesion model. 

## 2. Material and Methods

### 2.1. Light Source and Photosensitizer

The irradiation source was a diode laser (P-Laser; Panasonic Dental Co., Ltd., Osaka, Japan). Its wavelength, output power, and duty were 805 nm, 5 watts (W), and 20%, respectively, while the diameter of quartz optical fiber was 400 *μ*m. Indocyanine green (Opthagreen^®^; Santen Pharmaceutical Co., Ltd., Osaka, Japan) (12.5 mg/mL), a commonly used fluorescent fundus contrast medium, was used as the photosensitizer.

### 2.2. Antimicrobial Activity of Laser Irradiation

*E. faecalis* ATCC 29212 was cultured in brain-heart infusion (BHI) broth (Difco, Detroit, MI) at 37°C for 18 hours in an atmosphere of 5% CO_2_. The organisms were harvested by centrifugation at 10,000 ×g for 5 minutes then suspended in saline and adjusted to 3 × 10^6^  cells/mL using a spectrophotometer. Bacterial cell suspensions were mixed with sterilized saline or indocyanine green solution in sterilized test tubes for a final suspension of 1.5 × 10^6^ cells/mL, then individually irradiated by the diode laser at a distance of 1 mm for 30, 60, or 120 seconds. Following irradiation, each suspension (20 *μ*L including 3 × 10^2^ cells) was cultured on a BHI agar plate. The inoculated plates were incubated at 37°C for 18 hours in an atmosphere of 5% CO_2_ and colony-forming units (CFU) counted using the spread plate method.

### 2.3. Laser Irradiation Using *In Vitro* Model of Periapical Lesion Defect

**Figure 1:**
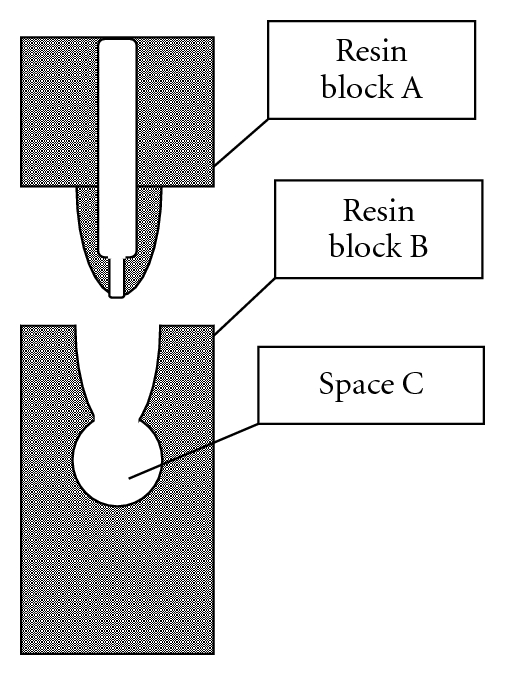
Schematic representation of present *in vitro* model of apical periodontitis. Resin block A, root part with a single root canal with an apical foramen (diameter 600 *μ*m). Resin block B, periapical tissue portion with a space (Space C, diameter 5 mm) fashioned as a periapical lesion defect.

An *in vitro* model of a periapical lesion defect was fashioned from resin blocks (Nissin Dental Products, Inc., Kyoto, Japan) (Figure 1). It was made up of a single root canal with an apical foramen (diameter; 600 *μ*m) in resin block A, which formed the root part of the model, while resin block B, which was used as the periapical tissue portion, had a space (Space C, diameter; 5 mm) fashioned as a periapical lesion defect. The model was sterilized before each experiment. *E. faecalis* was diluted to 2 × 10^6^ cells/mL with prewarmed (45°C) BHI broth containing 1% agar, then 20 *μ*L of the bacterial cell suspension was mixed with 20 *μ*L of distilled saline or indocyanine green solution in Space C (final concentration of *E. faecalis*, 1 × 10^6^  cells/mL). Resin block A was then inserted into resin block B and kept at room temperature. 

The optical fiber of the diode laser was inserted into the root canal to reach its apex then microorganisms in Space C were irradiated for 30, 60, or 120 seconds. In addition, the model was also irrigated with sterilized saline (4 mL) or 2.5% NaOCl (4 mL) for 120 seconds as negative and positive controls, respectively. After each treatment, *E. faecalis* in Space C were diluted 1/100 with sterilized saline and subjected to vortexing for 5 minutes, then 20 *μ*L of each sample (2 × 10^2^ cells) was cultured on a BHI agar plate. CFU were determined after incubation of the inoculated plates at 37°C for 18 hours in an atmosphere of 5% CO_2_.

### 2.4. Exposure of Microorganisms to Heat

*E. faecalis* suspensions were mixed with a sterilized indocyanine green solution (final suspension 1.5 × 10^6^  cells/mL) and incubated in a heat block (TERMO ALUMI BATH ALB-220: IWAKI GLASS Co., Ltd. Japan) at 65°C for 60 seconds, after which heat-treated *E. faecalis* (20 *μ*L; 3 × 10^2^  cells) were cultured on BHI agar plates. The inoculated plates were then incubated at 37°C for 18 hours in an atmosphere of 5% CO_2_ in air and CFU determined.

### 2.5. Temperature Monitoring during Laser Irradiation

Temperatures in the periapical lesion (Space C) and surrounding areas of the *in vitro* model were monitored during laser irradiation using infrared thermography (TH9100 WV: NEC Avio Infrared Technologies Co., Ltd. Japan). Just after performing laser irradiation, the inner temperature of the periapical lesion area (Space C) was also measured by immediate insertion of a thermocouple thermometer (Digital Thermometer CT-800: As One Corp., Japan).

### 2.6. Statistical Analysis

Differences among variables in the experiments were compared using Student's *t*-test.

## 3. Results

### 3.1. Antimicrobial Effects of Diode Laser with Photosensitizer and Disinfection of Periapical Lesion Defect in *In Vitro* Model

**Figure 2:**
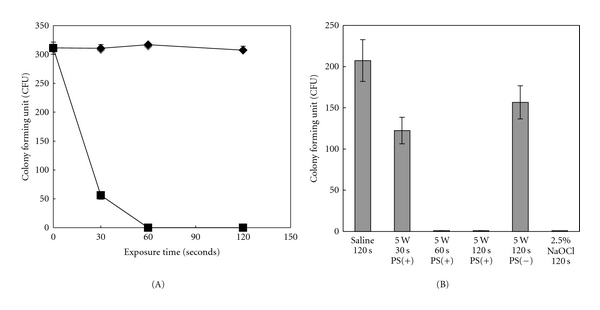
Antimicrobial effects of diode laser on *E. faecalis* in test tubes. The bacteria were exposed to laser irradiation with or without an added photosensitizer (closed diamond, without photosensitizer; closed square, with photosensitizer) for 30, 60, and 120 seconds. (A) Elimination of *E. faecalis* in model of apical periodontitis. *E. faecalis* in the presence and absence of a photosensitizer[PS(−), without photosensitizer; PS(+), with photosensitizer] in the lesion defect area of the model were exposed to diode laser irradiation for 30, 60, and 120 seconds. In addition, one sample was irrigated with sterilized saline for 120 seconds as a negative control (saline-120 s) and another with 2.5% NaOCl for 120 seconds as a positive control (2.5% NaOCl-120 s). (B) The number of viable cells after each treatment was counted. Data are expressed as the mean ± standard deviation of triplicate determinations. The experiment was performed three times, with similar results obtained in each.

There were no antimicrobial effects seen without the photosensitizer, whereas in its presence the cell viability of *E. faecalis* decreased to 72% after irradiation for 30 seconds and was not detected after irradiation of 60 seconds or more in test tubes (Figure 2(A)).

Figure 2(B) shows the antimicrobial effects of diode laser irradiation on *E. faecalis* in the *in vitro* model. Irrigation with saline did not have an antimicrobial effect, while that with NaOCl completely eliminated *E. faecalis*.When the diode laser was used without the photosensitizer for 120 seconds, the decrease in viability was only 25%. In contrast, a significant decrease in the viability of *E. faecalis* was observed following laser irradiation with the photosensitizer for 60 and 120 seconds.

### 3.2. Effects of Heat Produced by Laser Irradiation on Bacterial Cell Viability

**Figure 3:**
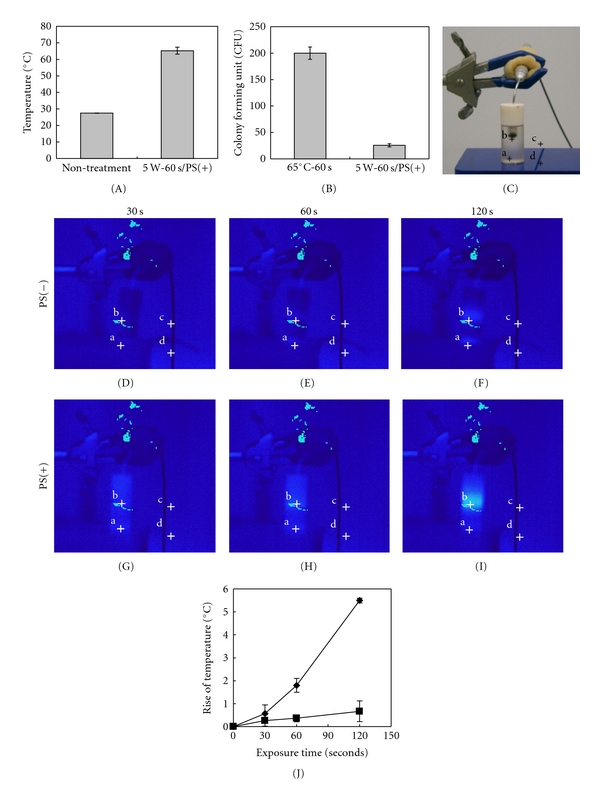
Effects of heat produced by laser irradiation. (A) Change of inner temperature in apical lesion area of *in vitro* model following laser irradiation. (B) Antimicrobial effects of heat on *E. faecalis*. The microorganisms were exposed to diode laser irradiation in the presence of a photosensitizer for 60 seconds [5 W-60 s/PS(+)] or exposed to heat at 65°C for 60 seconds (65°C-60 s). (C). Photograph of temperature change monitoring using a thermotracer, which was used to monitor 4 points (a, b, c, d) of the *in vitro* model. (D–F). Temperature change without photosensitizer. (G–I). Temperature change with photosensitizer. (J) Temperature change in periapical lesion area of the model during irradiation (closed diamond, with photosensitizer; closed square, without photosensitizer). The rise in temperature was calculated as the difference in temperature between points a and b. Data are expressed as the mean ± standard deviation of triplicate determinations. The experiment was performed three times, with similar results obtained in each.

The inner temperature of the apical lesion area of the *in vitro* model was determined with a thermocouple thermometer just after laser irradiation (Figure 3(A)). Irradiation for 60 seconds increased the temperature of the periapical lesion defect to 65°C. To analyze the cytotoxic effects of that elevated temperature (65°C) on *E. faecalis*, bacterial cells were incubated in a heat block at 65°C for 60 seconds (Figure 3(B)). There was no effect on bacterial cell viability by exposure to heating at 65°C, whereas the viability was significantly reduced when the bacterial cells were irradiated for 60 seconds in the presence of the photosensitizer. The rise in temperature in the periapical lesion and surrounding area of the *in vitro* model during laser irradiation was monitored using a thermotracer (Figure 3(C)). There was no change in temperature in either the lesion or surrounding area caused by irradiation when the photosensitizer was not added to the bacterial cell suspension (Figures 3(D)–3(F)). However, the thermotracer indicated an increase in temperature in the periapical lesion area with addition of the photosensitizer (Figures 3(G)–3(I)), which was 2°C after irradiation for 60 seconds and 6°C after that for 120 seconds (Figure 3(J)). In contrast, there was no rise in temperature observed in the area surrounding the lesion area even with addition of the photosensitizer (Figures 3(G)–3(I)).

## 4. Discussion

It is known that various photosensitizers are taken up by tumor cells and microorganisms, and those activated by laser irradiation interact with oxygen to produce radical species that have a toxic effect on those cells and microorganisms [25]. In the present study, indocyanine green, which was a photosensitizer commonly used as fluorescent fundus contrast medium that does not have toxicity toward surrounding tissues, increased the antimicrobial effects of laser irradiation in test tubes. However, that environment is different from that of a periapical lesion, which is surrounded by sound periapical tissues, and it takes many efforts to make a standardized apical lesion model *in vivo*.

 To simulate biological conditions, we prepared an *in vitro* apical lesion model and examined the antimicrobial effects of diode laser irradiation with or without the addition of a photosensitizer. When a bacterial cell suspension with indocyanine green was irradiated with the diode laser using periapical lesion model, *E. faecalis* CFU were significantly decreased to the same amount following irrigation with NaOCl. In a study of bactericidal activity against *E. faecalis* biofilm in extracted human teeth, it was reported that the total energy output of a diode laser was 36J [26]. In the present study, an irradiation time of 60 seconds was employed to achieve a total energy level of 76J and that with an added photosensitizer was adequate for a high level of sterilization. On the other hand, application of the present diode laser for 60 seconds increased the temperature of the lesion area to 65°C. However, exposure of *E. faecalis* to 65°C for 60 seconds in a heat block did not have any effects on bacterial viability. These results indicate that the reduction in viability of *E. faecalis* was from the laser irradiation itself, not from the heat produced by the irradiation, and that addition of a photosensitizer is essential for the observed antimicrobial effects.

 Adverse effects of intense heat produced by laser irradiation on sound periapical tissues are a concern in clinical applications. It has been reported that use of a diode laser resulted in the least amount of temperature increase among several types of lasers tested [19]. In the present study, it was found that irradiation at 5 W for 60 seconds in the presence of a photosensitizer raised it by 2°C in the lesion defect area, whereas no change in temperature was observed in the surrounding area. Furthermore, it has been demonstrated that the same diode laser irradiation with various levels of laser power (0.5 W to 5 W) for 2 minutes did not affect proliferation of mammalian cells *in vitro* and enhanced BMP-induced osteoblast differentiation by stimulating BMP/Smad signaling pathway [27]. Taken together, the present laser technique, which was adequate for elimination of *E. faecalis*, may have no cytotoxic effects on sound periapical tissues.

In conclusion, diode laser irradiation in combination with a photosensitizer had nearly the same antimicrobial effect as 2.5% NaOCl. Many clinicians prefer a diluted concentration to reduce the irritation potential of NaOCl, with 2.5% commonly recommended [28]. However, fibroblasts were found damaged by 2.5% NaOCl [3, 5, 28–30]. The present results indicated no cytotoxic effects from heat produced by laser irradiation, thus use of a diode laser with a photosensitizer is useful for treatment of periapical lesions without adverse effects on surrounding tissues. This standardized *in vitro* apical lesion model needs more improvement, but it may be useful for an endodontic study.
